# Effectiveness of comprehensive psychosocial interventions on quality‐adjusted life year and life expectancy in vascular dementia

**DOI:** 10.1002/alz70858_100023

**Published:** 2025-12-24

**Authors:** Mari Kasai, Kenichi Meguro, Geriatric Behavioral Neurology Research Group

**Affiliations:** ^1^ Tohoku University, Sendai, Japan; ^2^ Osaki‐Tajiri SKIP Center, Osaki, Japan

## Abstract

**Background:**

Quality‐adjusted life year (QALY) is a measure of quality of life used in health economic analyses of diseases, such as dementia. Psychosocial interventions for patients with Alzheimer's disease have been reported to affect their life expectancies and QALY (Meguro, 2014; Meguro, 2015). However, there have been no studies on the QALY of psychosocial interventions for vascular dementia (VaD). Here, we examined the effectiveness of comprehensive psychosocial interventions on QALY and life expectancies in patients with VaD.

**Method:**

The participants were 144 patients with VaD who were followed from the initial diagnostic visit to the memory clinic until death and analyzed retrospectively (Osaki‐Tajiri SKIP Center, from 1997 to 2016). VaD was diagnosed according to the NINDS‐AIREN criteria (Roman, 1993) for probable VaD (Meguro, 2014). All patients received adequate pharmacological treatment to control vascular risk factors, such as antithrombotic treatment. We divided the participants into four groups based on whether they received professional rehabilitation or were admitted to an institution. The QALY value for VaD was calculated using the estimated health state utility values for major, moderate, and minor strokes reported by Tengs (Pharmacoeconomics, 2003).

**Result:**

There were significant main effects of rehabilitation approach and nursing home residency, without interaction, on QALY and life expectancy between the day of the initial diagnostic visit and date of death (two‐way analysis of variance, *p* <0.05). The group of rehabilitation approach with nursing home residency had the highest QALY value and longest life expectancy compared to the other groups. After adjusting for age and sex, nursing home residency had a significant main effect on QALY and life expectancy, with a significant covariate effect on age (two‐way analysis of covariance, *p* <0.05).

**Conclusion:**

Although the effects of age must be considered for QALY and life expectancy, institutionalization and rehabilitation may be effective psychosocial interventions. In VaD, specialized psychosocial interventions may have a positive impact on quality of life and life expectancy.

